# High-Energy Diet and Shorter Light Exposure Drives Markers of Adipocyte Dysfunction in Visceral and Subcutaneous Adipose Depots of *Psammomys obesus*

**DOI:** 10.3390/ijms20246291

**Published:** 2019-12-13

**Authors:** Joanne T.M. Tan, Victoria A. Nankivell, Carmel Bilu, Tomer Shemesh, Stephen J. Nicholls, Paul Zimmet, Noga Kronfeld-Schor, Alex Brown, Christina A. Bursill

**Affiliations:** 1 South Australian Health & Medical Research Institute, Adelaide SA 5000, Australia; victoria.nankivell@sahmri.com (V.A.N.); tomer.shemesh@hotmail.com (T.S.); paul.zimmet@monash.edu (P.Z.); alex.brown@sahmri.com (A.B.); christina.bursill@sahmri.com (C.A.B.); 2 Adelaide Medical School, The University of Adelaide, Adelaide SA 5005, Australia; 3 School of Zoology, Tel Aviv University, Tel Aviv, Ramat Aviv 69978, Israel; carmel.bilu@gmail.com (C.B.); nogaks@tauex.tau.ac.il (N.K.-S.); 4 Monash Cardiovascular Research Centre, Monash University, Clayton VIC 3168, Australia; Stephen.Nicholls@monashhealth.org; 5 Department of Diabetes, Monash University, Clayton VIC 3800, Australia

**Keywords:** adipocyte differentiation, adipocyte hypertrophy, browning, circadian rhythms, inflammation, photoperiod

## Abstract

Dysfunctional adipose tissue phenotype underpins type 2 diabetes mellitus (T2DM) development. The disruption of circadian rhythms contributes to T2DM development. We investigated the effects of high-energy diet and photoperiod length on visceral and subcutaneous adipose tissue phenotype. *Psammomys obesus* sand rats exposed to neutral (12 light:12 dark) or short (5 light:19 dark) photoperiod were fed a low- (LE) or high- (HE) energy diet. The HE diet and/or short photoperiod reduced subcutaneous expression of adipocyte differentiation/function markers *C/ebpα*, *Pparδ*, *Pparγ* and *Adipoq*. Visceral *Pparα* levels were elevated in the 5:19HE group; however, the HE diet and/or short photoperiod decreased visceral *Pparγ* and *Adipoq* expression. 5:19HE animals had elevated *Ucp1* yet lower *Pgc-1α* levels. The HE diet increased visceral *Tgf-β1*, *Ccl2* and *Cd68* levels, suggestive of a pro-inflammatory state. Daily visceral rhythms of these genes were affected by a short photoperiod and/or HE diet. The 12:12HE, 5:19LE or 5:19HE animals had a higher proportion of larger adipocytes, indicating increased adipocyte hypertrophy. Collectively, the HE diet and/or shorter light exposure drives a dysfunctional adipose tissue phenotype. Daily rhythms are affected by a short photoperiod and HE diet in a site-specific manner. These findings provide mechanistic insight on the influence of disrupted circadian rhythms and HE diet on adipose tissue phenotype.

## 1. Introduction

Adipose tissue metabolism is closely linked to insulin resistance and variations in fat distribution are associated with T2DM [1]. Recent data suggest that both body fat distribution and adipocyte phenotype are more likely to increase fatal outcomes in obese patients than a higher general adiposity [2]. Adipose tissues vary in their impact on metabolic risk due to diverse gene expression profiles leading to differences in lipolysis and in the production and release of adipokines and cytokines. Furthermore, there are metabolic differences in specific adipose depots, with visceral adipose tissue shown to be less insulin-sensitive and more metabolically active than subcutaneous adipose tissue [3]. The development of T2DM is associated with dysfunctional adipose tissue phenotype, characterized by insulin-resistant adipocytes, adipocyte hypertrophy and a pro-inflammatory environment. Visceral adiposity and its white single-lipid-like adipocytes is known to exacerbate the development of T2DM. However, white adipocytes can transdifferentiate into beige/brown multi-mitochondrial-like adipocytes, forming a new type of thermogenic fat. Beige/brown adipocytes can oxidize glucose and lipids during UCP-1 mediated thermogenesis [4], which has the potential to reverse the diabetic milieu.

The circadian system or circadian clock is important for the synchronization of numerous physiological and metabolic processes within an organism with a periodic 24-h light/dark environmental cycle [5]. However, industrialization has led to a substantial shift in the timing of rest and activity phases, with an increasing number of employees working outside regular working hours [6]. Obesity has been related to irregular sleep/wake schedules attributed to the use of electric lighting which has expanded activities into the natural rest phase of diurnal humans [6,7]. Shift work has been shown to have significant negative health impacts, with numerous studies reporting an association of shift work with elevated risks for overweight, metabolic syndrome and T2DM [8,9,10,11]. Increasing evidence shows that disruption of circadian rhythms increases the risk of many chronic diseases including T2DM [12,13] and diurnal rhythms in the white adipose tissue transcriptome are disturbed in obese individuals with T2DM [14]. Animal models of chronodisruption that include scheduled food access, high fat diet, mistimed activity, sleep disruption and light exposure all show significant impacts on the development of obesity, metabolic syndrome, impaired glucose metabolism and hyperglycemia [6]. Circadian clock genes are known to influence adipocyte tissue physiology. In particular, the period protein ‘PER2′ is found to modulate the activity of PPARγ, the critical transcription factor that drives adipocyte differentiation [15].

The *Psammomys obesus* sand rat, a gerbil, is a unique polygenic rodent model that resembles the phenotypic and pathophysiological development of T2DM in humans. This highly relevant model develops T2DM without the use of pharmacological treatments to destroy beta cells such as streptozotocin [16]. In its native desert environment, *P. obesus* subsists on a diet of saltbush and remains lean and normoglycemic [17]. A proportion of these animals spontaneously and naturally develop the quintessential characteristics that underpin T2DM when fed a diet that contains a higher energy content [18]. In addition, sand rats are diurnal, which, unlike the nocturnal *Mus musculus*, makes them a valuable model for modelling diurnal humans and to investigate the outcomes of disrupting circadian rhythms. A recent study suggested that T2DM development in this species is caused by circadian disruption, spontaneously developed under laboratory conditions, which is manifested when the animals are fed a high-energy diet. Moreover, daily rhythms in behaviour and physiology were disrupted in animals exposed to a short photoperiod, accelerating T2DM development. Therefore, the sand rat is an excellent diurnal model for the study of the connection between circadian disruption and the development of T2DM [19]. To date, the effect of circadian rhythm disruption on adipose tissue phenotype has not been investigated in *P. obesus*. This study sought to determine whether high-energy diet, in combination with a disrupted circadian rhythm, contribute to gene and phenotypic changes reflective of dysfunctional adipose tissue including insulin-resistant adipocytes, a pro-inflammatory milieu and adipocyte hypertrophy. We hypothesized that short photoperiod would exacerbate the detrimental effects of the high-energy diet, driving an insulin-resistant and pro-inflammatory adipose tissue phenotype.

We found that a high-energy diet and short photoperiod drives features characteristic of a dysfunctional adipose tissue phenotype. This includes elevated pro-inflammatory marker expression and increased adipocyte hypertrophy, which is associated with the development of T2DM. Short photoperiod and high-energy diet strikingly affected daily rhythms in the visceral adipose depot, a key contributor to whole body insulin resistance. Taken together, our study provides further mechanistic insight on the influence of disrupted circadian rhythms and high-energy diet on adipose tissue phenotype.

## 2. Results

### 2.1. Daily Rhythms of Adipogenesis Markers are Differentially Expressed in Visceral Adipose Tissue with Changes in Photoperiod and Diet

**Figure 1:**
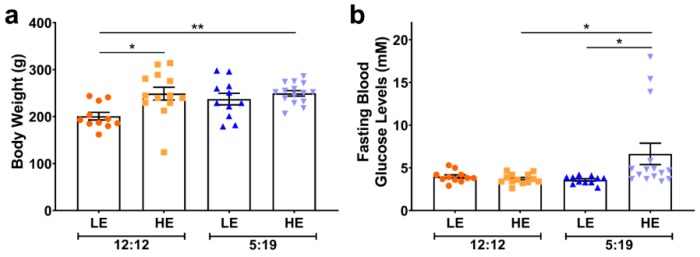
Body weight and fasting blood glucose levels are influenced by short photoperiod and high-energy diet. (**a**) Body weight and (**b**) Fasting blood glucose levels are expressed as mean ± SEM (*n* = 11–15/group). * *p* < 0.05, ** *p* < 0.01 by two-way ANOVA (Tukey’s multiple comparisons post hoc). Photoperiod denoted by light:dark hours 12:12, neutral; 5:19, short; LE, low-energy diet; HE, high-energy diet.

Animals fed the high-energy (HE) diet significantly gained more weight irrespective of photoperiod compared to the 12:12LE group (Figure 1a). Furthermore, animals fed the HE diet and exposed to the short photoperiod (5:19HE) had elevated fasting blood glucose levels compared to both the 12:12HE and 5:19LE groups (Figure 1b).

**Figure 2:**
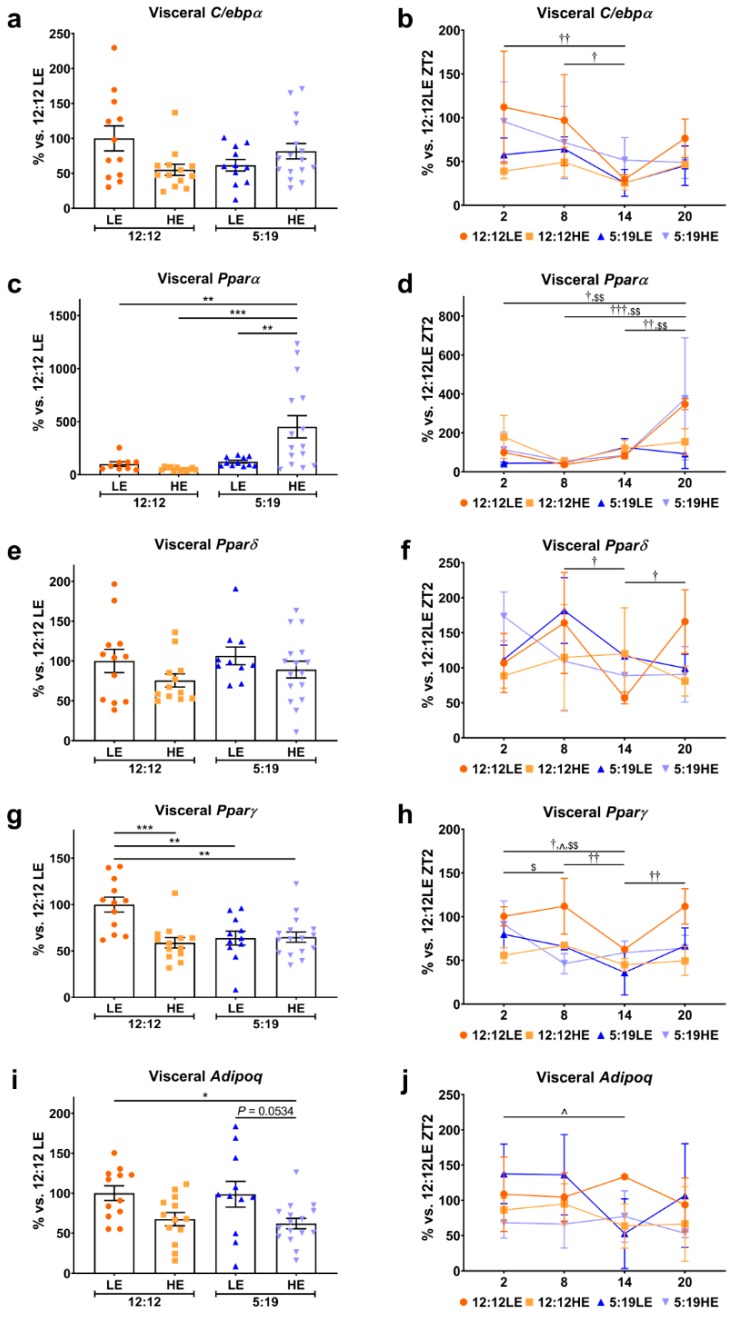
Daily rhythms of adipogenesis markers are differentially expressed in visceral adipose tissue with changes in photoperiod and diet. Visceral adipose expression of adipogenesis/metabolic dysfunction markers (**a**,**b**) *C/ebpα*, (**c**,**d**) *Pparα*, (**e**,**f**) *Pparδ*, (**g**,**h**) *Pparγ* and (**i**,**j**) *Adipoq*. Average gene expression was determined across the four groups and normalized using the ^ΔΔ^*Ct* method to *Cyclophilin* and to the 12:12LE group (mean ± SEM, *n* = 11 – 15/group, left panels). Daily rhythms were determined by measuring gene expression based on euthanasia time points (i.e., ZT2, ZT8, ZT14 and ZT20) and normalized using the ^ΔΔ^*Ct* method to *Cyclophilin* and the 12:12LE group euthanized at ZT2 (mean ± SD, *n* = 2–6/group, right panels). * *p* < 0.05, ** *p* < 0.01, *** *p* < 0.001; ^†^ *p* < 0.05, ^††^ *p* < 0.01, ^†††^ *p* < 0.001 within 12:12LE group; ^^^ *p* < 0.05 within 5:19LE group; ^$^ *p* < 0.05, ^$$^ *p* < 0.01 within 5:19HE group by two-way ANOVA (Tukey’s multiple comparisons post hoc). Photoperiod denoted by light:dark hours 12:12, neutral; 5:19, short; LE, low-energy diet; HE, high-energy diet.

Increasing evidence suggests that numerous metabolic functions of adipose tissues are regulated by the circadian clock to achieve temporal coordination and whole body homeostasis [20]. We therefore sought to determine if HE diet and/or photoperiod length influenced adipocyte dysfunction in a site-specific manner; the average expression and daily rhythm of key mediators involved in adipogenesis were assessed. The average expression of the transcription factor *C/ebpα*, a critical driver of adipocyte differentiation, was not different in the visceral depots across all four groups (Figure 2a). Interestingly, while the 12:12LE animals showed a significant reduction of *C/ebpα* expression at ZT14, these effects were not observed with HE diet and short photoperiod (Figure 2b). We also observed differential expression of the three PPAR transcription isoforms. Visceral average expression of *Pparα*, which regulates adipocyte lipid absorption, was strikingly elevated in the 5:19HE animals when compared to the 12:12LE, 12:12HE and 5:19LE groups (Figure 2c). *Pparα* levels were increased in both 12:12LE and 5:19HE groups at ZT20 (Figure 2d). Visceral *Pparδ*, which controls lipid metabolism, did not show any differences in the average daily expression (Figure 2e); however, only the 12:12LE group showed a daily *Pparδ* rhythm (Figure 2f). Visceral *Pparγ*, the key transcription factor that drives adipogenesis, was reduced in animals fed the HE diet, exposed to short photoperiod or both when compared to the 12:12LE control animals (Figure 2g). Assessment of daily rhythms showed significant time point differences with the 12:12LE, 5:19LE and 5:19HE groups (Figure 2h). The adipokine adiponectin is only expressed in mature fully differentiated adipocytes and has been recognized as a key regulator of insulin sensitivity and tissue inflammation [21,22]. Only the 5:19HE animals had lower average visceral *Adipoq* expression when compared to 12:12LE control group (Figure 2i). Daily *Adipoq* rhythm was only observed in the 5:19LE group (Figure 2j).

### 2.2. Daily Rhythms of Adipogenesis Markers Are Differentially Expressed in Subcutaneous Adipose Tissue with Changes in Photoperiod and Diet

**Figure 3:**
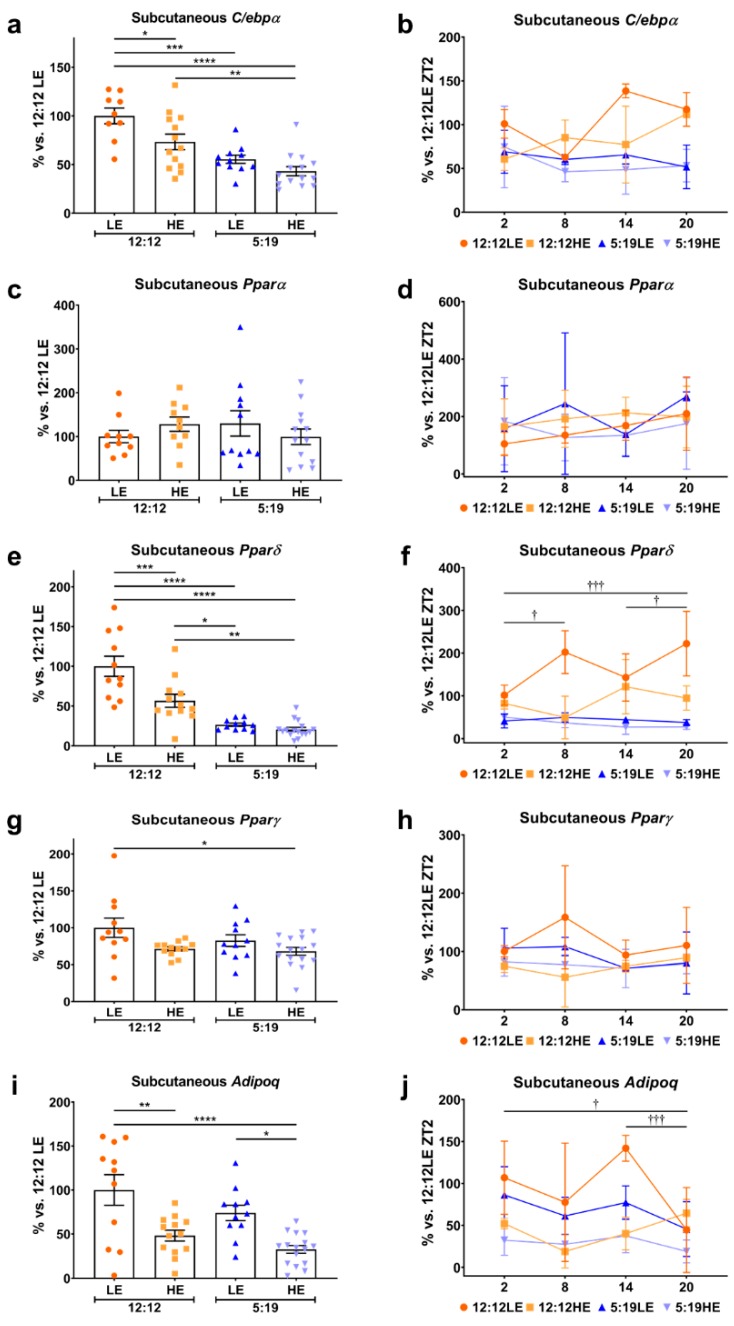
Daily rhythms of adipogenesis markers are differentially expressed in subcutaneous adipose tissue with changes in photoperiod and diet. Subcutaneous adipose expression of adipogenesis/metabolic dysfunction markers (**a**,**b**) *C/ebpα*, (**c**,**d**) *Pparα*, (**e**,**f**) *Pparδ*, (**g**,**h**) *Pparγ* and (**i**,**j**) *Adipoq*. Average gene expression was determined across the four groups and normalized using the ^ΔΔ^*Ct* method to *Cyclophilin* and to the 12:12LE group (mean ± SEM, *n* = 11–15/group, left panels). Daily rhythms were determined by measuring gene expression based on euthanasia time points (i.e., ZT2, ZT8, ZT14 and ZT20) and normalized using the ^ΔΔ^*Ct* method to *Cyclophilin* and the 12:12LE group euthanized at ZT2 (mean ± SD, *n* = 2–6/group, right panels). * *p* < 0.05, ** *p* < 0.01, *** *p* < 0.001, **** *p* < 0.0001; ^†^ *p* < 0.05, ^†††^ *p* < 0.001 within 12:12LE group by two-way ANOVA (Tukey’s multiple comparisons post hoc). Photoperiod denoted by light:dark hours 12:12, neutral; 5:19, short; LE, low-energy diet; HE, high-energy diet.

Similar observations were also seen in the average daily expression and daily rhythms in the subcutaneous adipose depot. In comparison to the 12:12LE control group, subcutaneous *C/ebpα* was reduced in the 12:12HE animals (Figure 3a), which was further exacerbated in animals exposed to short photoperiod in a stepwise fashion for 5:19LE then 5:19HE, however, no daily rhythm differences were observed between time points across all the groups (Figure 3b). Subcutaneous *Pparα* levels were not different between groups (Figure 3c,d). In contrast to visceral *Pparδ*, animals exposed to the short photoperiod expressed lower levels of subcutaneous *Pparδ* with a moderate decrease observed in animals fed the HE diet alone (Figure 3e). Comparisons of the daily *Pparδ* rhythm showed significant time point differences in the 12:12LE group (Figure 3f). Only the dual combination of the short photoperiod and HE diet significantly reduced average subcutaneous *Pparγ* expression compared to the 12:12LE control animals (Figure 3g), with no distinct differences seen in daily rhythms (Figure 3h). Subcutaneous average *Adipoq* levels decreased with HE diet which was exacerbated with a short photoperiod (Figure 3i) with only the 12:12LE group showing significant daily rhythm changes (Figure 3j).

### 2.3. High-Energy Diet and Short Photoperiod Influences Daily Rhythm Expression of Browning Markers

**Figure 4:**
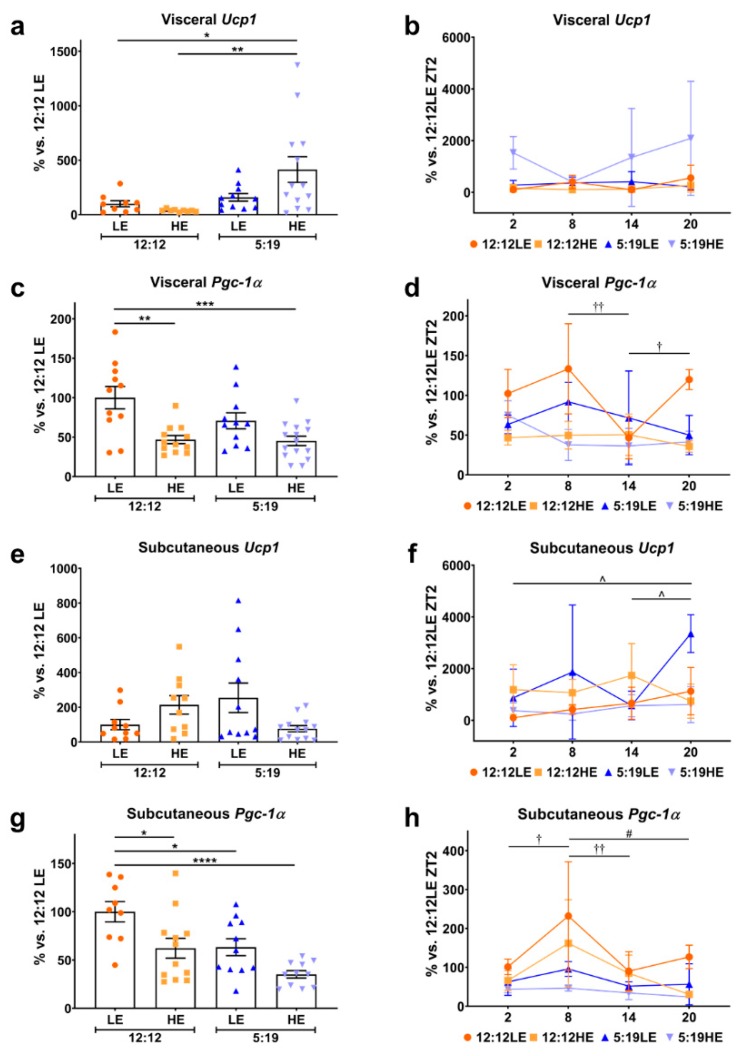
High-energy diet and short photoperiod influences daily rhythm expression of browning markers. Expression of visceral (**a**,**b**) *Ucp1*, (**c**,**d**) *Pgc-1α*; and subcutaneous (**e**,**f**) *Ucp1*, (**g**,**h**) *Pgc-1α*. Average gene expression was determined across the four groups and normalized using the ^ΔΔ^*Ct* method to *Cyclophilin* and to the 12:12LE group (mean ± SEM, *n* = 11–15/group, left panels). Daily rhythms were determined by measuring gene expression based on euthanasia time points (i.e., ZT2, ZT8, ZT14 and ZT20) and normalized using the ^ΔΔ^*Ct* method to *Cyclophilin* and the 12:12LE group euthanized at ZT2 (mean ± SD, *n* = 2–6/group, right panels). * *p* < 0.05, ** *p* < 0.01, *** *p* < 0.001, **** *p* < 0.0001; ^†^ *p* < 0.05, ^††^ *p* < 0.01 within 12:12LE group; ^#^ *p* < 0.05 within 12:12HE group; ^^^ *p* < 0.05 within 5:19LE group by two-way ANOVA (Tukey’s multiple comparisons post hoc). Photoperiod denoted by light:dark hours 12:12, neutral; 5:19, short; LE, low-energy diet; HE, high-energy diet.

There has been increasing interest in the ability of white adipocytes to transdifferentiate into beige/brown adipocytes, which are then able to oxidize glucose and lipids during thermogenesis, providing an alternate therapeutic avenue for metabolic diseases [4,23]. To determine whether HE diet and/or photoperiod had any effect on adipose tissue browning, two browning markers, *Ucp1* and *Pgc-1α* were measured (Figure 4). Interestingly, the average expression of visceral *Ucp1* was increased in the 5:19HE animals compared to the 12:12 cohorts (Figure 4a); however, no differences were seen with the daily rhythms (Figure 4b). Animals fed the HE diet had lower average visceral *Pgc-1α* levels (Figure 4c). Significant changes in daily visceral *Pgc-1α* rhythms were observed in the 12:12LE group (Figure 4d). While no differences were observed in average subcutaneous *Ucp1* (Figure 4e), the 5:19LE group had a significantly different daily rhythm (Figure 4f). Subcutaneous *Pgc-1α* levels were lower in animals that were either fed the HE diet or exposed to a short photoperiod, with the most striking decrease seen in the combination of both (Figure 4g). Interestingly, only the 12:12LE and 12:12HE groups showed significant differences in the daily rhythm of subcutaneous *Pgc-1α* (Figure 4h).

### 2.4. High-Energy Diet Drives Inflammation in Visceral Adipose Tissue

**Figure 5:**
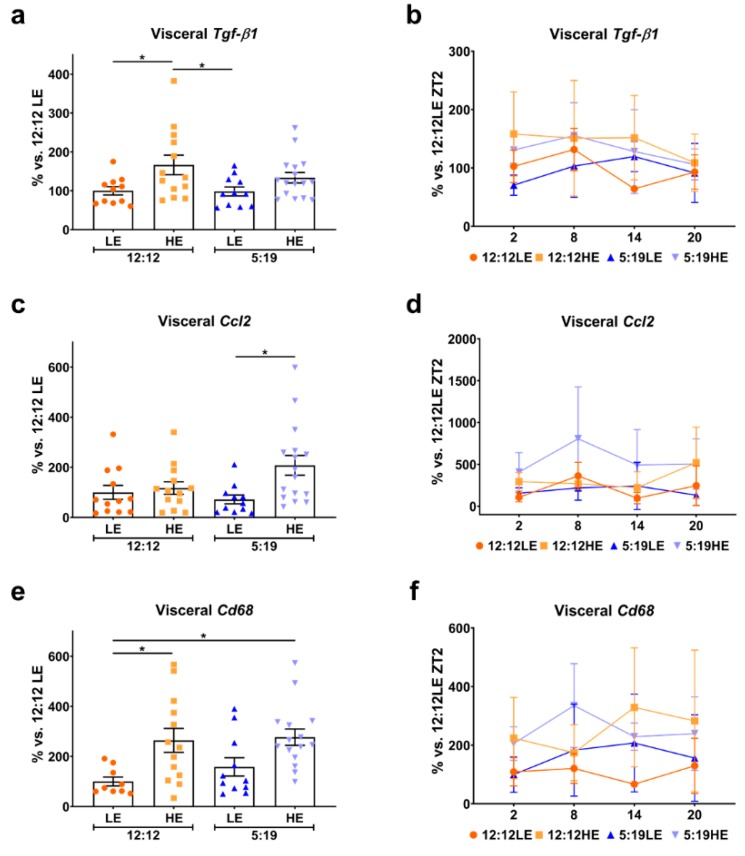
High-energy diet drives inflammation in visceral adipose tissue. Visceral adipose expression of (**a**,**b**) *Tgf-β1*, (**c**,**d**) *Ccl2* and (**e**,**f**) *Cd68*. Average gene expression was determined across the four groups and normalized using the ^ΔΔ^*Ct* method to *Cyclophilin* and to the 12:12LE group (mean ± SEM, *n* = 11−15/group, left panels). Daily rhythms were determined by measuring gene expression based on euthanasia time points (i.e., ZT2, ZT8, ZT14 and ZT20) and normalized using the ^ΔΔ^*Ct* method to *Cyclophilin* and the 12:12LE group euthanized at ZT2 (mean ± SD, *n* = 2–6/group, right panels). * *p* < 0.05 by two-way ANOVA (Tukey’s multiple comparisons post hoc). Photoperiod denoted by light:dark hours 12:12, neutral; 5:19, short; LE, low-energy diet; HE, high-energy diet.

Chronic low-grade inflammation is known to contribute to adipocyte dysfunction. Excessive lipid accumulation promotes the secretion of inflammatory cytokines such as TGF-β1 and CCL2 by adipocytes, which, in turn, promote the infiltration and expansion of macrophages, perpetuating the inflammatory cycle [24,25]. To assess whether HE diet and/or photoperiod contributed to any increase in inflammation, expression of *Tgf-β1, Ccl2* and the macrophage marker *Cd68* were measured. The HE diet augmented average visceral *Tgf-β1* levels in animals exposed to the normal photoperiod when compared to their LE diet counterparts (Figure 5a), with no differences observed in the daily rhythms of all four groups (Figure 5b). The HE diet only increased average visceral *Ccl2* levels in the 5:19HE group when compared to 5:19LE (Figure 5c), with this group showing the highest daily amplitude (Figure 5d). An increase in average visceral *Cd68* levels was only observed in the HE-fed animals (Figure 5e). Assessment of daily rhythms showed marked variations across all groups (Figure 5f).

**Figure 6:**
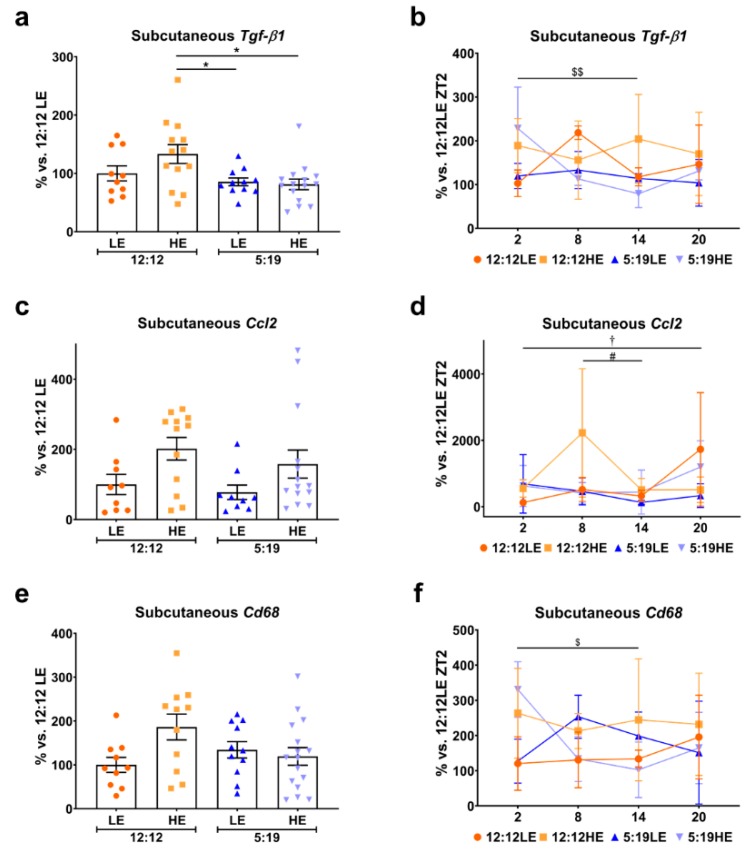
Expression of inflammatory markers in subcutaneous adipose tissue. Subcutaneous adipose expression of (**a**,**b**) *Tgf-β1*, (**c**,**d**) *Ccl2* and (**e**,**f**) *Cd68*. Average gene expression was determined across the four groups and normalized using the ^ΔΔ^*Ct* method to *Cyclophilin* and to the 12:12LE group (mean ± SEM, *n* = 11–15/group, left panels). Daily rhythms were determined by measuring gene expression based on euthanasia time points (i.e., ZT2, ZT8, ZT14 and ZT20) and normalized using the ^ΔΔ^*Ct* method to *Cyclophilin* and the 12:12LE group euthanized at ZT2 (mean ± SD, *n* = 2–6/group, right panels). * *p* < 0.05; ^†^ *p* < 0.05 within the 12:12LE group; ^#^ *p* < 0.05 within the 12:12HE group; ^$^ *p* < 0.05, ^$$^ *p* < 0.01 within the 5:19HE group by two-way ANOVA (Tukey’s multiple comparisons post hoc). Photoperiod denoted by light:dark hours 12:12, neutral; 5:19, short; LE, low-energy diet; HE, high-energy diet.

In the subcutaneous adipose tissue, animals exposed to the short photoperiod had significantly lower average subcutaneous *Tgf-β1* levels compared to the 12:12HE group (Figure 6a). Daily subcutaneous *Tgf-β1* rhythms were varied across the groups (Figure 6b). No differences were observed in subcutaneous *Ccl2* average expression (Figure 6c); however, significant changes in daily rhythms were observed for the 12:12 groups (Figure 6d). While no differences were detected with average subcutaneous *Cd68* levels (Figure 6e), large variations in daily rhythms were observed (Figure 6f), similarly to that seen with visceral *Cd68*.

### 2.5. Daily Rhythms of Per2 in Visceral and Subcutaneous Adipose Depots

**Figure 7:**
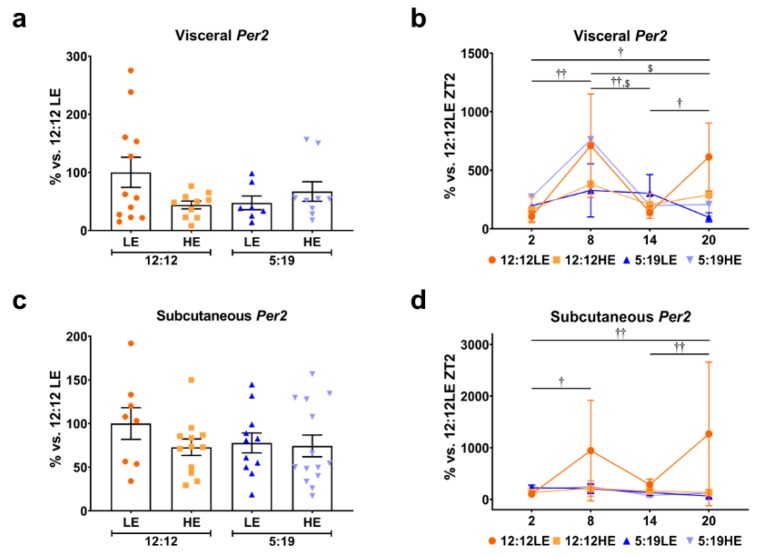
Daily rhythms of *Per2* in visceral and subcutaneous adipose depots. Expression of (**a**,**b**) visceral and (**c**,**d**) subcutaneous *Per2*. Average gene expression was determined across the 4 groups and normalized using the ^ΔΔ^*Ct* method to *Cyclophilin* and to the 12:12LE group (mean ± SEM, *n* = 11 − 15/group, left panels). Daily rhythms were determined by measuring gene expression based on euthanasia time points (i.e., ZT2, ZT8, ZT14 and ZT20) and normalized using the ^ΔΔ^*Ct* method to *Cyclophilin* and the 12:12LE group euthanized at ZT2 (mean ± SD, *n* = 2–6/group, right panels). ^†^ *p* < 0.05, ^††^ *p* < 0.01 within 12:12LE group; ^$^ *p* < 0.05 within 5:19HE group by two-way ANOVA (Tukey’s multiple comparisons post hoc). Photoperiod denoted by light:dark hours 12:12, neutral; 5:19, short; LE, low-energy diet; HE, high-energy diet.

Finally, we assessed the average expression and daily rhythms of the established clock gene *Per2*. No significant differences were observed in the average expression of visceral and subcutaneous *Per2* across all four groups irrespective of diet or photoperiod (Figure 7a,c). Interestingly, daily visceral *Per2* rhythms were strikingly different in the 12:12LE and 5:19HE groups (Figure 7b). However, only the 12:12LE group had strikingly different daily subcutaneous *Per2* rhythms. (Figure 7d).

### 2.6. High-Energy Diet Induces Morphological Changes in Adipose Depots

**Figure 8:**
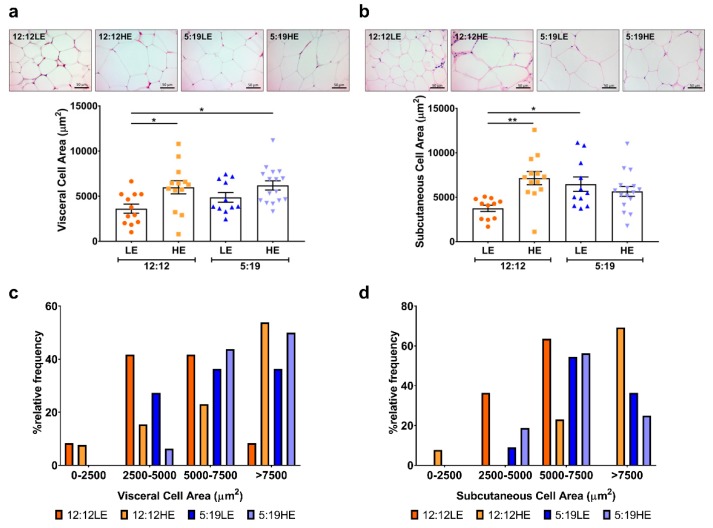
High-energy diet induces morphological changes in adipose depots. Representative images and corresponding average (**a**) visceral and (**b**) subcutaneous cell area calculated from average cell area measurement/animal per group. Data expressed as mean ± SEM (*n* = 11–15/group). * *p* < 0.05, ** *p* < 0.01 by two-way ANOVA (Tukey’s multiple comparisons post hoc). (**c**) Visceral and (**d**) subcutaneous cell area measured was plotted as a relative frequency distribution. Photoperiod denoted by light:dark hours 12:12, neutral; 5:19, short; LE, low-energy diet; HE, high-energy diet.

Due to changes in the expression of genes involved in the control of adipogenesis, we next measured morphological changes in the adipocytes in both visceral and subcutaneous depots to assess adipocyte hypertrophy. Of the animals exposed to the normal photoperiod there was a significant increase in the area of visceral (Figure 8a,c) and subcutaneous (Figure 8b,d) adipocytes when fed the HE diet compared to the low diet control group. Visceral adipocytes were also larger in animals that were exposed to the short photoperiod in combination with the high-energy diet. In contrast, exposure to the short photoperiod alone (5:19LE) resulted in larger subcutaneous adipocytes compared to the 12:12LE group (Figure 8b). For both visceral and subcutaneous depots, animals exposed to the HE diet, short photoperiod or both, exhibited a higher proportion of larger adipocytes (> 7500 μm^2^) (Figure 8c,d).

## 3. Discussion

The role of the adipose tissue in the pathophysiology of T2DM remains to be fully elucidated. Adipose tissue regulation is highly complex. Therefore, a range of factors need to be considered including: the different fat pads in distinct anatomical locations, the many different types of fat, as well as their ability to interchange into other types of fat cells or even de-differentiate altogether [26]. Development of T2DM is associated with dysfunctional adipose tissue phenotype, characterized by increased adipocyte size and a pro-inflammatory response. Increasing evidence shows that disruption of circadian rhythms contributes to disease progression, and it was recently found that daily rhythms in the white adipose tissue transcriptome are disturbed in individuals with T2DM compared with lean individuals [14]. In this study we have used the *Psammomys obesus* sand rat, a unique animal model that mimics T2DM development in humans. We found that a short photoperiod, which causes circadian disruption [19], in combination with the HE diet (1) drives changes in the daily rhythms of markers of adipogenesis, inflammation and browning, (2) increases a dysfunctional adipose tissue phenotype and (3) triggers a pro-inflammatory environment—key characteristics that drive T2DM. This highlights the suitability of the *Psammomys obesus* sand rat as a model that depicts the molecular mechanisms that drive T2DM and provides significant advantages over current monogenic mouse and rat models of disease [27]. Significant metabolic differences have been reported in specific adipose depots, with visceral adipose shown to be less insulin sensitive and more metabolically active than subcutaneous adipose. In this study, we found that a short photoperiod in combination with an HE diet disrupts daily rhythms of genes that drive a dysfunctional adipose tissue phenotype, and this varied distinctly between visceral and subcutaneous adipose depots.

Adipocyte differentiation is a critical process that involves the transition of an undifferentiated fibroblast-like preadipocyte into mature lipid-laden adipocytes. Previous studies have found that visceral adipocytes are less insulin sensitive and less able to fully differentiate into mature adipocytes compared to subcutaneous adipocytes [28], indicating that impaired adipocyte differentiation occurs in a site-specific manner. C/EBPα and PPARγ are integral drivers of adipocyte differentiation [29,30]. Co-expression of C/EBPα and PPARγ has synergistic effects on adipogenic conversion in vitro [31]. We find that C/EBPα and PPARγ expression are driven by HE diet, short photoperiod or the combination of both in a site-dependent manner; with *C/ebpα* levels being strikingly inhibited in the subcutaneous adipose tissue while in the visceral adipose tissue, *Pparγ* was reduced, suggesting that adipocyte differentiation is impaired and driven by different transcription factors under these conditions. It has been previously reported that ChIP-chip analysis of C/EBPα and PPARγ binding showed that 60% of the genes regulated in adipocyte differentiation bind to both factors simultaneously, whereas 3% of the genes are bound by PPARγ only and 25% by C/EBPα alone [32]. This suggests that the adipose site-specific regulation of C/EBPα and PPARγ would not have an impact on overall impaired adipocyte differentiation as both regulate largely overlapping sets of gene targets. This is supported by the concurrent inhibition of adiponectin (*Adipoq*), an insulin-sensitizing adipocytokine that is only expressed in mature, fully differentiated adipocytes. In both adipose depots, the reduction in adiponectin is primarily driven by the HE diet, irrespective of circadian disruption. This suggests that high caloric intake is more important in driving adipocyte differentiation and function.

Recent studies have reported the influence of clock genes on adipocyte differentiation, with the circadian gene PER2 shown to directly regulate the transcriptional activity of PPARγ and functions as a critical regulator of lipid metabolism and adipogenesis [15]. Daily average *Per2* levels showed similar expression patterns to that seen with *Pparγ*, suggesting that variations in circadian rhythm may have had a direct influence on adipogenesis. However, this did not quite reach statistical significance and perhaps other factors (such as HE diet) may be stronger contributors to adipocyte differentiation and function. Interestingly, when the groups were sub-divided according to different collection time points, *Pparγ* and *Per2* levels showed similar trends in both adipose depots. This suggests that the daily rhythm of both *Pparγ* and *Per2* may, to a certain extent, influence adipose tissue phenotype. Consistently with this, PPARγ has previously been shown to exhibit a circadian expression pattern that is magnified by high-fat diet consumption [33]. We also found that other gene targets involved in adipogenesis, inflammation and browning had diurnal rhythms with differential effects seen in the visceral and subcutaneous adipose depots of the 12:12LE groups. Strikingly, in many cases, the amplitude of gene changes is much smaller and even non-existent in the 5:19 groups, providing further evidence on the growing impact of circadian disruptions on T2DM development [34]. Our subcutaneous adipose findings are consistent with a recent paper that reported daily clock and metabolic gene expression rhythms are decreased in subcutaneous adipose tissue of obese individuals with T2DM compared with lean participants [14]. However, our data are unique in showing that HE diet and short photoperiod can also influence daily rhythms in the visceral adipose depot. Given the small sample size per group, further studies are required to fully elucidate the effects of diurnal rhythms on adipose tissue phenotype.

PPARα, which regulates lipoprotein metabolism and fatty acid oxidation [35], has also been shown to induce the formation of beige/brown adipocytes [36]. Visceral adiposity and its white single-lipid-like adipocytes has long been shown to exacerbate the progression of T2DM. However, under specific stimuli, white adipocytes can transdifferentiate into beige/brown multi-mitochondrial-like adipocytes, forming a new type of thermogenic fat. Beige/brown adipocytes can oxidize glucose and lipids during UCP-1 mediated thermogenesis [4], which has the potential to reverse the diabetic milieu. Interestingly, we observed a striking increase in visceral *Pparα* concomitant with an increase in visceral *Ucp1* in the 5:19HE group, suggesting that the visceral adipocytes may be transdifferentiating into beige/brown adipocytes. We observed no differences in *Pparα* or *Ucp1* levels in the subcutaneous depot, again highlighting a site-specific effect. PPARγ is also known to modulate brown adipocyte function by interacting with its transcriptional coactivator PGC-1α [35]. Animals fed the HE diet had lower levels of *Pgc-1α* in both adipose depots, which paralleled the low levels of *Pparγ*. Given that PGC-1α is essential for brown fat thermogenesis but not brown fat differentiation [37], these findings suggest that the transdifferentiated beige adipocytes may not exert beneficial thermogenic functions that could reverse the effects of a HE diet.

PPARδ exerts powerful regulatory functions on adipose tissue metabolism and energy homeostasis [35]. The PPARδ agonist GW501516 lowered plasma triglyceride levels in obese monkeys while raising high-density lipoprotein levels [38]. Furthermore, transgenic PPARδ expression in adipose tissue produced lean mice that are resistant to obesity, hyperlipidemia and tissue steatosis, induced genetically or by a high fat diet [39]. Compared to non-obese subjects, there was a decrease in PPARδ levels in subcutaneous adipose tissues of morbidly obese subjects [40]. Consistent with this, subcutaneous *Pparδ* levels were suppressed in HE-fed animals and was further exacerbated by a short photoperiod.

Adipose tissue inflammation is a key mechanism that underpins the pathogenesis of obesity-linked insulin resistance, as evidenced by experimental animal models and clinical studies [41,42]. The ability of adipocytes to express a suite of inflammatory cytokines have reaffirmed the notion that obesity is considered a state of chronic low-grade inflammation [25]. TGF-β1 is a cytokine with a dual facet in obesity-related inflammation. In the adipose tissue of obese subjects, TGF-β1 release is inhibited by pro-inflammatory cytokines and enhanced by dexamethasone, a potent anti-inflammatory drug that antagonises those cytokines [43]. Conversely, TGF-β1 promotes accumulation of macrophages, collagen deposition and remodelling in fat tissue of obese mice [44]. Rats fed a high fat diet had increased levels of TGF-β mRNA in epididymal and retroperitoneal visceral fat depots [45]. Consistently with this, we observed an increase in visceral *Tgf-β1* levels in animals fed the HE diet, with a similar trend seen in subcutaneous adipose. However, in this study, *Tgf-β1* levels were lower in animals exposed to a short photoperiod, suggesting circadian rhythm disruptions may influence this regulation. In support of this, a recent study showed that BMAL1, an essential clock transcription activator, inhibits brown adipogenesis via direct transcriptional control of key components of the TGF-β pathway, including TGF-β1 [46].

Inflammatory changes in adipose tissue are driven in part by the infiltration of adipose tissue macrophages, which secrete a host of cytokines including CCL2, leading to a chronic state of inflammation strongly implicated in mechanisms of metabolic dysregulation [47]. Depot-specific differences in inflammatory function have been reported, with the visceral adipose tissue exhibiting increased expression of inflammatory markers and macrophage infiltration compared to subcutaneous adipose tissue [48,49]. Consistently with this, we found that animals fed the HE diet had elevated levels of the macrophage marker *Cd68*, in the visceral adipose tissue, suggestive of increased macrophage infiltration. However, only the 5:19HE animals had elevated visceral *Ccl2* levels.

Adipocyte hypertrophy is a strong established indicator of the degree of metabolic impairment/disturbance in patients [50,51]. Numerous studies have reported that increased adipocyte size is associated with key characteristic parameters that underpin T2DM, including insulin resistance and a pro-inflammatory phenotype [52,53]. Comparison studies of the two adipose depots showed that only visceral adipocyte size was related to metabolic health, increased macrophage recruitment and overall whole-body insulin resistance [54,55]. In this study, we found that animals fed a HE diet had increased visceral adipocyte size irrespective of photoperiod while subcutaneous adipocytes were larger in animals that were either on the HE diet or short photoperiod alone. This suggests that the HE diet may have a greater effect on visceral adipocyte size than a change in photoperiod. It is also possible that disruption in circadian rhythms could increase the susceptibility to develop T2DM and that HE diet facilitated this development in these animals.

## 4. Materials and Methods

### 4.1. Animal Studies

We obtained samples from an experiment performed on HsdHu Diabetes Prone male sand rats (*Psammomys obesus*, 6–7 months old). Prior to the start of the experiment, all animals were maintained on a low-energy diet (Koffolk Ltd., Tel Aviv, Israel) and tested for body weight and glucose tolerance. Animals were assigned to experimental groups based on the weights and blood glucose levels to avoid baseline bias. All experimental procedures followed the NIH guidelines for the care and use of laboratory animals and were approved by the Institutional Animal Care and Use Committee (IACUC) of Tel Aviv University (Permit Number: L15055). Adult male sand rats (*n* = 11–15/group) were exposed to the following conditions: 1) normal photoperiod, low-energy diet (12:12LE); 2) normal photoperiod, high-energy diet (12:12HE); 3) short photoperiod, low-energy diet (5:19LE) and 4) short photoperiod, high-energy diet (5:19HE). Animals in the LE groups were fed a special low-energy diet (14% protein, 1.7% fat, 15.4% Crude fiber; Product No.: 1078; Koffolk Ltd., Israel), which is the caloric equivalent to their native diet. Animals in the HE groups were fed the standard rodent diet (21% protein, 4% fat, 4% Crude fiber; Product No.: 2018; Koffolk Ltd.), which contains a higher caloric density that is known to drive T2DM development in sand rats [56]. On week 10, the sand rats were weighed then euthanized at four different time points (with six-hour intervals, ZT, 2, 8, 14, 20, [ZT 0 = lights on]) and a sample from their visceral and subcutaneous adipose tissue was collected and snap-frozen for further analysis.

### 4.2. Gene Expression Analysis

Quantitative real-time PCR was performed for *Pparα*, *Pparδ*, *Pparγ*, *Pgc-1α*, *Tgf-β1*, *Ccl2*, *Cd68*, *Per2* and *Cyclophilin* using primers designed previously [19,57,58,59]. Primers were designed for *C/ebpα* (F: 5′-TCGGTGGACAAGAACAGCAA-3′, R: 5′-CGTTGCGCTGTTTGGCTTTA-3′), *Adipoq* (F: 5′-CCCCAATGTCCCATTCGCTT-3′, R: 5′-GAACGGCCTTGTCCTTCTTG-3′) and *Ucp1* (F: 5′-GGCATTCAGAGGCAAATCAGC-3′, R: 5′-CTTGCTTCCAAAGAGGCAGGTG-3′) by aligning *Homo sapiens*, *Mus musculus* and *Rattus norvegicus* sequences and generated based on the overlapping consensus sequences using Primer Blast. Relative changes in average gene expression were determined across the 4 groups irrespective of the time point of euthanasia and normalized using the ^ΔΔ^*Ct* method to *Cyclophilin* and to the 12:12LE control group. Given that animals within each group were euthanized at six-hour intervals from ZT0 (lights on), the daily rhythm was determined by measuring gene expression based on euthanasia time points (i.e., ZT2, ZT8, ZT14 and ZT20) and normalized using the ^ΔΔ^*Ct* method to *Cyclophilin* and the 12:12LE group euthanized at ZT2.

### 4.3. Determination of Adipocyte Size

5 μm visceral and subcutaneous adipose tissue sections were stained with either Masson’s Trichrome (Sigma) or Haemotoxylin and Eosin (Thermofisher, Waltham, MA, USA) to define adipocyte morphology. Two sections per animal were selected and two fields of view were imaged per section with an AxioLab microscope attached to a camera (Zeiss, Oberkochen, Baden-Württemberg, Germany) at 400× total magnification. Area of adipocytes was determined by manual tracing of the interior of the adipocyte and results analyzed as (1) mean area per animal and (2) distribution of adipocyte size in both depots using ZEN lite 2.3.

### 4.4. Statistical Analysis

Gene expression data expressed as either averaged daily levels (mean ± SEM, *n* = 11–15/group) or daily rhythms (mean ± SD, *n* = 2–6/group). Differences between treatment groups or time points within groups were calculated using a two-way ANOVA with Tukey’s multiple comparisons post hoc test. Significance was set at a two-sided *p* < 0.05.

## 5. Conclusions

**Figure 9:**
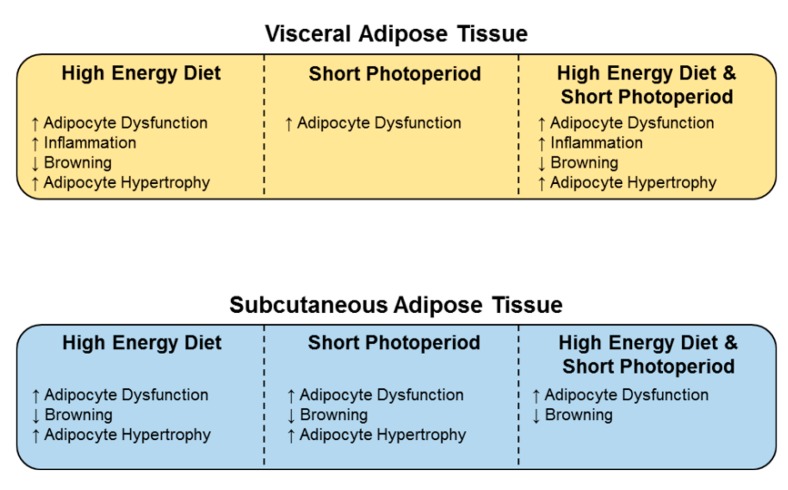
High-energy diet and/or shorter light exposure drives adipocyte hypertrophy and dysfunction coupled with a pro-inflammatory milieu, key characteristics that drive T2DM development. Summary of the effects of high-energy diet, short photoperiod or the combination of both on key markers involved in adipocyte dysfunction, inflammation and browning. Differential effects were observed between visceral and subcutaneous adipose depots, suggesting that the mechanisms that underpin T2DM development may be dependent on the adipose site.

In conclusion, we find that the *Psammomys obesus* is a unique animal model that resembles the human pathogenesis of T2DM. It therefore provides clinically relevant insight into the molecular mechanisms that influence adipocyte dysfunction and the predisposition to T2DM. Our studies highlight that high-energy diet and/or shorter light exposure drives adipocyte hypertrophy and dysfunction coupled with a pro-inflammatory milieu (Figure 9). These are all key characteristics that drive the development of T2DM. Furthermore, we observed differential effects in daily rhythms between visceral and subcutaneous adipose, suggesting that the mechanisms that underpin T2DM development may be dependent on the adipose site. These findings provide further understanding into the mechanisms that drive a dysfunctional adipose tissue phenotype and the influence of high-energy diet and disrupted circadian rhythms on the development of T2DM.

## Abbreviations

Adipoq	Adiponectin
C/ebpα	CCAAT/enhancer binding protein α
Ccl2	C-C motif chemokine ligand 2
HE	High energy
LE	Low energy
Per2	Period 2
PGC-1α	PPAR gamma coactivator-1α
PPAR	Peroxisome proliferator-activated receptor
TGF-β1	Transforming growth factor-β1
Ucp1	Uncoupling protein 1
